# Er-Chen Decoction Alleviates High-Fat Diet-Induced Nonalcoholic Fatty Liver Disease in Rats through Remodeling Gut Microbiota and Regulating the Serum Metabolism

**DOI:** 10.1155/2022/6221340

**Published:** 2022-03-31

**Authors:** Jing Miao, Liying Guo, Huantian Cui, Li Wang, Bo Zhu, Jinyan Lei, Peng Li, Jianwei Jia, Zhaiyi Zhang

**Affiliations:** ^1^Tianjin Second People's Hospital, Tianjin, China; ^2^Shandong Provincial Key Laboratory of Animal Cell and Developmental Biology, School of Life Sciences, Shandong University, Qingdao, China; ^3^Tianjin University of Traditional Chinese Medicine, Tianjin, China

## Abstract

Many studies have found that the dysfunction in gut microbiota and the metabolic dysfunction can promote nonalcoholic fatty liver disease (NAFLD) development. Er-Chen decoction (EC) can be used in the treatment of NAFLD. However, the mechanism of this hepatoprotection is still unknown. In this study, we constructed a rat model with NAFLD fed with high-fat chow and administered EC treatment. The therapeutic effects of EC on NAFLD were evaluated by measuring transaminases, blood lipid levels, and pathological changes in the liver. In addition, we measured the effects of EC on liver inflammatory response and oxidative stress. The changes in gut microbiota after EC treatment were studied using 16S rRNA sequencing. Serum untargeted metabolomics analysis was also used to study the metabolic regulatory mechanisms of EC on NAFLD. The results showed that EC decreased the serum transaminases and lipid levels and improved the pathological changes in NAFLD rats. Furthermore, EC enhanced the activities of SOD and GSH-Px and decreased MDA level in the liver. EC treatment also decreased the gene and protein levels of IL-6, IL-1*β*, and TNF-*α* in the liver and serum. The 16S rRNA sequencing and untargeted metabolomics indicated that EC treatment affected the gut microbiota and regulated serum metabolism. Correlation analysis showed that the effects of EC on taurine and hypotaurine metabolism, cysteine and methionine metabolism, and vitamin B6 metabolism pathways were associated with affecting in the abundance of *Lactobacillus*, *Dubosiella*, *Lachnospiraceae, Desulfovibri, Romboutsia, Akkermansia, Intestinimonas*, and *Candidatus_saccharimonas* in the gut. In conclusion, our study confirmed the protective effect of EC on NAFLD. EC could treat NAFLD by inhibiting oxidative stress, reducing inflammatory responses, and improving the dysbiosis of gut microbiota and the modulation of the taurine and hypotaurine metabolism, cysteine and methionine metabolism, and vitamin B6 metabolism pathways in serum.

## 1. Introduction

Nonalcoholic fatty liver disease (NAFLD) is a hepatopathy syndrome characterized by steatosis and fat accumulation in hepatocytes [1]. Recently, the NAFLD incidence has increased, and it has replaced viral hepatitis as the most common chronic liver disease worldwide, with a prevalence of 25% in adults [2]. NAFLD could also develop into type 2 diabetes, liver cirrhosis, and liver cancer in severe cases, thus, posing a great threat to human life and health, and a major economic burden to patients, families, and society [3]. Currently, the major treatments for NAFLD are exercise therapy and pharmacological therapy; however, the acceptance and sustainability of exercise therapy are still lacking [4]. For pharmacological therapy, lipid-lowering drugs (e.g., simvastatin), such as insulin inhibitors (e.g., pioglitazone), and antioxidants (e.g., vitamin E) are generally used [5–7]. However, some lipid-lowering drugs possess potential hepatotoxicity, and some of them fail to decrease hepatic fat deposition and may even aggravate liver injury [8, 9]. Therefore, the development of traditional Chinese medicines with lipid-lowering and hepatoprotective effects would be more appropriate for treating NAFLD and have wide application prospects as well as practical significance.

Recently, gut microbiota has been widely studied as influential factors closely related to NAFLD. Gut microbiota primarily influence NAFLD development by altering the microecological composition of the gut, suppressing the expression of inflammatory factors, and regulating metabolites, such as short-chain fatty acids, bile acids, and endogenous ethanol [10]. Some probiotics, such as *Bifidobacteria*, *Lactobacillus*, and *Bacteroidetes* have essential roles in maintaining gut microbiota balance and treating NAFLD. Intestinal *Bifidobacteria* decrease intestinal cholesterol absorption by degrading bile salts and promoting fecal bile acid excretion, which affects inhibiting cholestasis and enhancing enterohepatic circulation [11]. Metabolomics allows systematic identification and quantification of metabolite levels and can elucidate the disease pathogenesis and the mechanism of drug action on metabolic levels [12]. Metabolite levels are impacted by the gut microbiota and can directly show the current metabolic state of an organ or a cell [13]. Studies have discovered that traditional Chinese medicine (TCM) has outstanding advantages in enhancing dyslipidemia in NAFLD and other aspects and has wide application value [14, 15]. Shenling Baizhu powder alleviates NAFLD through its antioxidant, anti-inflammatory, lipid-lowering, and gut microbiota regulatory effects [16–18]. Dachaihu decoction enhances the alteration of gut microbiota and regulates arachidonic acid (AA), glycerophospholipid, and glycine/serine/threonine metabolic pathways to increase metabolic disorders in NAFLD model rats [19]. Using 16S rRNA sequencing combined with metabolomics can elucidate the mechanism of action of Chinese herbal formulas through the interaction between gut microbiota and host metabolism.

Er-Chen decoction (EC), composed of *Pinellia ternata* (Thunb.) Makino, *Citrus* *×* *aurantium* L., *Smilax glabra* Roxb., and *Glycyrrhiza uralensis* Fisch. ex DC., and has been using in the treatment of NAFLD [20]. Experimental studies have indicated that EC can significantly decrease blood lipid levels, improve insulin resistance, and decrease body weight in rats on a high-fat diet [21]. Clinical observation has also discovered that EC modified formula significantly improves fat deposition, lowers blood lipids, and improves patients' quality of life in NAFLD patients [22]. However, its specific action mechanism has not been elucidated.

To better understand these mechanisms, a rat model of NAFLD was generated and the therapeutic effects of EC on NAFLD rats were evaluated, and then the effects of EC on inflammation and oxidative stress in NAFLD rats were examined. The changes in gut microbiota after EC treatment were investigated using 16S rRNA sequencing. In addition, the metabolic regulatory mechanism of EC on NAFLD was also analyzed using an untargeted metabolomics approach.

## 2. Materials and Methods

### 2.1. Reagents

Simvastatin (C_25_H_38_O_5_, molecular weight: 418.57 Da, CAS No.: 79902-63-9) was obtained from Sigma-Aldrich Co., Ltd. (USA). High-fat chow (10% lard, 5% sugar, 4% cholesterol, 0.2% propylthiouracil, 0.5% sodium cholate, and 80.3% basal chow) was purchased from Beijing Sibeifu Bioscience Co., Ltd. (Beijing, China). Aspartate aminotransferase (AST), alanine aminotransferase (ALT), triglyceride (TG), total cholesterol (TC), superoxide dismutase (SOD), methane dicarboxylic aldehyde (MDA), and glutathione peroxidase (GSH-Px) test kits were purchased from Nanjing Jiancheng Biological Engineering Institute (Nanjing, China). Oil Red O Staining kit was obtained from Solarbio Biotechnology Co., Ltd. (Beijing, China). Total RNA isolation, cDNA reverse transcription, quantitative polymerase chain reaction (qPCR) kits, and primers for qPCR experiment were purchased from TianGen Biotechnology Co., Ltd. (Beijing, China). Enzyme-linked immunosorbent assay (ELISA) kits of rat interleukin (IL)-1*β*, IL-6, and tumor necrosis factor-alpha (TNF-*α*) were purchased from Shanghai BlueGene Biotech Co., Ltd. (Shanghai, China).

### 2.2. Preparation of Er-Chen Decoction

15 g of *Pinellia ternata* (Thunb.) Makino, 15 g of *Citrus* *×* *aurantium* L., 9 g of *Smilax glabra* Roxb., and 4.5 g of *Glycyrrhiza uralensis* Fisch. ex DC were weighed out, and eight times the volume of water was added for 30 min of decoction and concentrated into 2 g crude herb/mL.

### 2.3. Animals

Specific-pathogen-free (SPF)-grade male Sprague Dawley (SD) rats aged 6–8 weeks and weighing (200 ± 20) g were provided by Beijing Huafukang Biotechnology Co., Ltd. The housing environment was maintained at 25 ± 2°C with a relative humidity of 50 ± 15% and a 12 h light-dark cycle with access to water and food *ad libitum*. The experiment was approved by the Ethics Committee of Nankai University.

### 2.4. Animal Grouping

After one week of adaptation rearing, the NAFLD rat model was replicated as described in the literature [23]. Fifty rats were randomly divided into control, model, simvastatin, EC low-dose, and EC high-dose groups (*n* = 10 per group). The rats in control group were fed with normal chow, and the rats in remaining four groups were given high-fat chow (4% cholesterol, 10% lard, 5% sugar, 0.5% sodium cholate, 0.2% propylthiouracil, and 80.3% basal chow), all for 12 weeks. During the 12 weeks of model establishment, drug interventions were administered simultaneously, where the control and the model groups were given 2 mL/rat of saline daily through intragastric administration. The simvastatin group was administered 1.8 mg/kg/d of simvastatin through intragastric administration [24], and the EC low and EC high-dose groups were given 4.5 g/kg/d and 9 g/kg/d (2 mL/rat) of EC through intragastric administration based on the clinically equivalent dose in humans, all for 12 weeks. The body weight of rats was recorded every two weeks during the experiment.

### 2.5. Serum Biochemical Indicator Test

After modeling and drug administration, rats in each group were fasted for 24 h and anesthetized by intraperitoneal injection of 1% phenobarbital (10 mL/kg). Blood was collected from the abdominal aorta and centrifuged at 4°C and 3,000 r/min for ten minutes to separate the serum. Serum levels of ALT, aspartate transaminase (AST), triglyceride (TG), and total cholesterol (TC) were measured using reagent kits.

### 2.6. Liver Pathological Staining

Immediately after model establishment and drug administration, the liver tissue from the same part of the left lobe of the liver was eliminated, cleaned, fixed in 10% formalin, and dehydrated for paraffin embedding. The tissue was cut into 5 *μ*m sections using a microtome (model RM2125 RTS, bought from Leica), routinely stained using HE, cleared, mounted, and then observed under a light microscope (model ECLIPSE E100, bought from Nikon) for pathological alterations in the liver. The pathological severity of steatosis, lobular inflammation, and hepatocyte ballooning was determined using our previously published NAFLD activity score (NAS) [25].

In addition, 10 *μ*m-thick frozen liver tissues were cut using a cryotome (model CM3050 S, purchased from Leica) and fixed on slides to dry at room temperature for 5 min. The tissue was stained using the Oil Red O staining kit manufacturer's instructions and then immediately observed under a light microscope. The percentage of the stained area of Oil Red O was determined using Image-Pro Plus 6.0.

### 2.7. Antioxidant Indicator Test of the Liver Tissue

A 100 mg of liver tissues were weighed and immersed in 900 *μ*L saline, homogenized by ultrasonication, and centrifuged at 2,000 r/min for 10 min; the supernatant was obtained. The levels of SOD, GSH-Px, and MDA in liver tissue homogenates were detected according to the manufacturer's instructions.

### 2.8. ELISA for Serum Inflammatory Factor Expression

The expression levels of the cytokines TNF-*α*, IL-1*β*, and IL-6 in the serum of each group were measured using ELISA. The specific methods are as described in the kit's manufacturer's instructions.

### 2.9. Detection of mRNA Expression of the Liver Inflammatory Factor Using qPCR

Total RNA was isolated from frozen liver tissue based on the established protocol of the kits. After testing RNA purity and concentration, the RNA was reverse transcribed into cDNA, and qPCR was conducted to detect the mRNA expression of *TNF-α* (primer sequence Forward: GAGCACGGAAAGCATGATCC; Reverse: TAGACAGAAGAGCGTGGTGG), *IL-1β* (primer sequence Forward: GGGATGATGACGACCTGCTA; Reverse: TGTCGTTGCTTGTCTCTCCT), and *IL-6* (primer sequence Forward: CTCATTCTGTCTCGAGCCCA; Reverse: TGAAGTAGGGAAGGCAGTGG) in the liver tissue. The relative expression of mRNA was computed following the relative quantification method using *β-actin* (primer sequence Forward: TCTTCCAGCCTTCCTTCCTG; Reverse: CACACAGAGTACTTGCGCTC) as an internal control. Quantification was conducted based on the 2^−△△CT^ method.

### 2.10. 16S rRNA Sequencing

#### 2.10.1. Fecal Genomic DNA Extraction

The total genomic DNA of rat cecum contents was obtained using the CTAB/SDS method, and concentration and purity of DNA samples were measured using a 1% agarose gel.

#### 2.10.2. PCR Amplification and Sequencing of 16S rRNA Gene

The 16S rRNA gene (V3 to V4 region) was amplified using universal primers 338F and 806R. The primer sequences of 338F and 806R were 5′-ACTCCTACGGGAGGCAGCAG-3′ and 5′-GGACTACHVGGGTWTCTAAT-3′, respectively. The PCR system consisted of 10 ng template DNA, 0.2-*µ*M primers (forward and reverse, respectively), and 15 *μ*L Phusion® High-Fidelity PCR Master Mix (New England Biolabs, Ipswich, Massachusetts, United States). The reaction conditions were predenaturation at 98°C for 1 min, denaturation at 95°C for 10 s, annealing at 50°C for 30 s, extension at 72°C for 30 s, with 15 cycles. In the end, the reaction was held at 72°C for 5 min, and then the reaction was stored at 4°C. Then, Qiagen Gel Extraction Kit (Qiagen, Germany) was used to purify the mixed PCR products. TruSeq® DNA PCR-Free Sample Preparation Kit (Illumina, USA) was used to generate the sequencing libraries, and Qubit@ 2.0 Fluorometer (Thermo Scientific) and Agilent Bioanalyzer 2100 system were used to access the library quality. Finally, 250 bp of paired-end sequences were obtained based on the Illumina NovaSeq platform.

#### 2.10.3. Sequencing Data Analysis

Raw sequencing data were assembled using a FLASH (v1.2.7) [26], and quality control was applied to the sequence to acquire the effective tags. The tags were clustered using Uparse software (Uparse v7.0.1001) [27] at 97% similarity level. Then, the operational taxonomic units (OTUs) were obtained. The Mothur algorithm-based Silva database [28] was used to annotate the OTUs based on taxonomic information. MUSCLE software was used for multiple sequence comparisons (v3.8.31) [29]. Alpha diversity index and beta diversity analysis were subsequently conducted. Differences between groups in diversity indices were analyzed using Wilcoxon Rank Sum test, and the Kruskal–Wallis rank sum test was used (Games-Howell was selected as the posthoc test), combined with the false discovery rate of multiple testing methods to screen for differential microbiota, and differences with *P* < 0.05 were considered statistically significant. Finally, Phylogenetic Investigation of Communities by Reconstruction of Unobserved States database (PICRUSt) analysis was conducted to predict the relevant gene pathways that may be influenced by each group of differential microbiota.

### 2.11. Metabolomic Analysis

#### 2.11.1. Serum Sample Processing

A 100 *μ*L serum sample was added to 400 *μ*L 80% methanol, vortexed and shaken, placed on an ice bath for 5 min, and centrifuged for 20 min (15,000 g at 4°C). After centrifugation, the supernatant was diluted using ultrapure water to 53% methanol, followed by centrifugation at 15,000 g at 4°C for 20 min, and the supernatant was collected as the testing sample. An equal amount of each sample was mixed and used as the quality control (QC) sample. Throughout the whole analysis, periodic analysis was conducted to monitor the stability of the instrument.

#### 2.11.2. Chromatographic and Mass Spectrometric Conditions

The chromatography was conducted on a Hypesil Gold column (C18) and chromatographic column (1.9 *μ*m, 2.1 mm × 100 mm) with a mobile phase comprising (A) 0.1% formic acid and (B) methanol, using a gradient elution at 0 min, 98% A, 2% B; 1.5 min, 98% A, 2% B; 12 min, 0% A, 100% B; 14 min, 0% A, 100% B; 14 min, 0% A, 100% B; 14.1 min, 98% A, 2% B; and 17 min, 98% A, 2% B. The column temperature was 40°C, the flow rate was 0.2-mL/min, and the injection volume was 2 *μ*L. The mass spectrometry conditions were simultaneously detected in positive and negative ion mode based on the electrospray ionization (ESI) source. The ESI setting source were as follows: spray voltage: 3.2 kV; aux gasflow rate: 10-arb; sheath gas flow rate: 40-arb; capillary temp: 320°C. Polarity: positive; negative; scanning range: 100–1500 m/z. Throughout the experiment, QC was added after every six samples to analyze the stability of the experiment.

#### 2.11.3. Data Processing

Molecular characteristic peaks were found based on mass spectrometry detection technique. The molecular peaks were identified using the combination of mzCloud, mzVault, and MassList databases. Data preprocessing of the raw files (.raw) obtained from mass spectrometry was conducted based on Compound Discoverer 3.1 (CD3.1, Thermo Fisher) software. Molecular formulas were then predicted according to the molecular ion peaks and fragment ions and compared with MassList, mzCloud, and mzVault databases, thus identifying the metabolites. Metabolites with a coefficient of variance less than 30% [30] in QC samples were then retained as final identifications for subsequent analysis. The peaks found in the samples were integrated using CD3.1 software to obtain the quantitative results of the metabolites. The data were then subjected to QC to ensure the reliability and accuracy of the data. Next, multivariate statistical analysis (principal component analysis (PCA) and orthogonal partial least squares discriminant analysis (OPLS-DA)) of the metabolites was conducted to show differences in metabolic patterns among various groups. Metabolite correlation analysis was used to show the relationship between samples and metabolites. Finally, the biological significance of metabolite correlation was explained through functional analysis, such as metabolic pathway. Metabolites (*P* < 0.05 and variable importance of projection (VIP) > 1, fold change (FC) > 1.25 or FC < 0.80) were screened as the differential metabolites. Metabolic pathway enrichment analysis of differential metabolites was conducted based on KEGG platform. Finally, pathways with *P* < 0.05 and impact >0.1 were selected as the enriched pathways.

#### 2.11.4. Statistical Processing

Statistics and analysis were conducted using SPSS 20.0 statistical software, and data are expressed as $\bar{x}\pm s$. One-way analysis of variance was used for comparing groups. Differences with *P* < 0.05 were considered statistically significant.

## 3. Results

### 3.1. Therapeutic Effects of EC on NAFLD Rats

**Figure 1:**
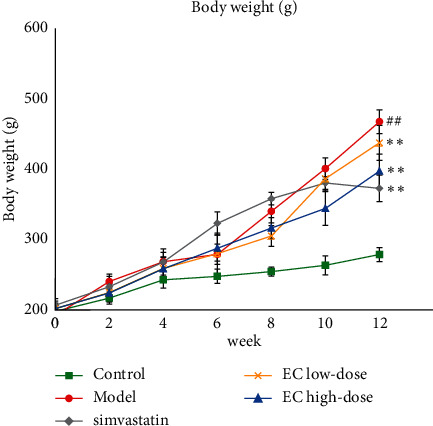
EC treatment decreases the body weight in NAFLD model rats. Control, model, simvastatin, EC-low dose, and EC-high dose (*n* = 10 per group) groups are shown. Data are reported as the mean ± SD. ^##^*P* < 0.01 compared with the control group; ^*∗∗*^*P* < 0.01 compared with the model group.

**Figure 2:**
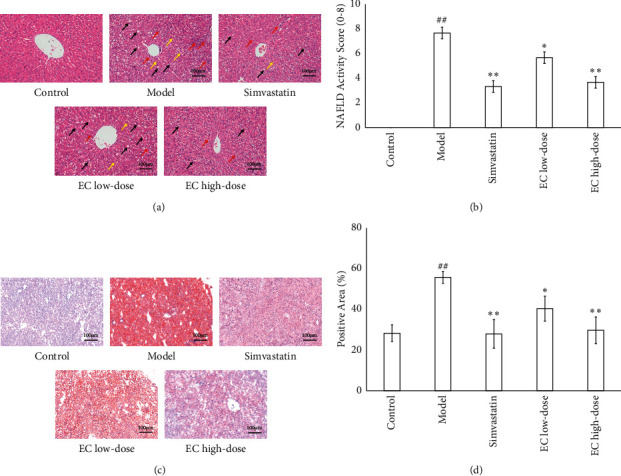
EC treatment improved the hepatosteatosis in NAFLD model rats. (a, b) H&E staining indicated that EC treatment ameliorated the pathological changes of the liver in NAFLD rats. Black arrows indicate the steatosis of hepatocytes, red arrows indicate lobular inflammation, and yellow arrows hepatic cord disorder (200x). (c, d) Oil Red O staining shows that EC treatment reduced the lipid accumulation in the liver (200x). Control, model, simvastatin, EC low-dose, and EC high-dose (*n* = 10 per group) groups are shown. Data are reported as the mean ± SD. ^##^*P* < 0.01 compared with the control group; ^*∗*^*P* < 0.05 compared with the model group; ^*∗∗*^*P* < 0.01 compared with the model group.

Compared with the control group, the body weight of rats in the model group was significantly increased (*P* < 0.01, Figure 1), and the low and high-doses of EC and simvastatin treatment significantly decreased the body weight of rats in the NAFLD model compared with the model group (*P* < 0.01, respectively, Figure 1). As indicated by HE staining, the liver of the control rats was structurally intact, and their hepatocytes were neatly arranged in a radial pattern. The hepatocytes in the portal region and around the central vein were clear and intact, and the nuclei were uniform in size and shape and located in the center of the hepatocytes. In the liver of rats in the model group, many hepatocytes indicated steatosis, where the hepatocytes were distinctively swollen and rounded; the nuclei were squeezed to one side; the cytoplasm was filled with many fat vacuoles; lipid droplets were of various sizes, which were even fused into huge lipid droplets; and inflammatory cell infiltration was observed. The EC high-dose treatment and simvastatin treatment significantly alleviated steatosis and inflammatory cell infiltration induced by high lipid chow (Figure 2(a)). Similarly, the NAS score was significantly higher in the model group than in the control group, and H&E staining in the positive control and EC groups exhibited a lower NAS score than in the model group (*P* < 0.01, Figure 2(b)). The results of Oil Red O staining proposed that the nuclei of hepatocytes in the control rats were blue, and no red lipid droplets appeared in the hepatocytes. The lipid levels in the liver tissue of rats in the model group were significantly increased compared to that in the control group (*P* < 0.01). Compared to the model group, the lipid accumulation in the hepatocytes of rats in all the dosing groups was decreased (*P* < 0.01, respectively) (Figures 2(c) and 2(d)). In addition, the results of serum biochemical indicators indicated that the serum TC and TG levels as well as ALT and AST were significantly increased in the model group compared to those in the control group (*P* < 0.01, respectively); compared to the model group, simvastatin and EC high-dose intervention significantly decreased the levels of TC levels (*P* < 0.01, *P* < 0.05, respectively), TG levels (*P* < 0.01, respectively), and the activities of ALT (*P* < 0.05, respectively), and AST (*P* < 0.01, respectively) in serum (Table 1).

### 3.2. Anti-Inflammatory and Antioxidative Effects of EC on NAFLD Rats

**Figure 3:**
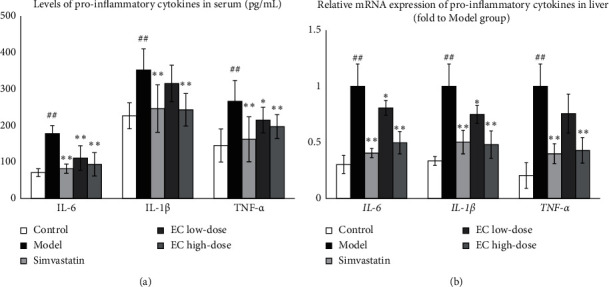
EC treatment reduced the inflammatory response in NAFLD model rats: (a) The levels of *IL-6*, *IL-1β*, and *TNF-α* in serum were decreased after EC treatment. (b) EC treatment downregulated the mRNA expression of *IL-6*, *IL-1β*, and *TNF-α* in the liver. Control, model, simvastatin, EC low-dose, and EC high-dose (*n* = 10 per group) groups are shown. Data are reported as the mean ± SD. ^##^*P* < 0.01 compared with the control group; ^*∗*^*P* < 0.05 compared with the model group; ^*∗∗*^*P* < 0.01 compared with the model group.

ELISA was used to detect the levels of proinflammatory factors IL-6, IL-1*β*, and TNF-*α* in the serum of rats in each group to analyze the effect of EC on the inflammatory response of NAFLD model rats. Compared to the control group, the serum levels of proinflammatory factors IL-6, IL-1*β*, and TNF-*α* were significantly increased in the model group (*P* < 0.01, respectively); compared to the model group, the interventions of simvastatin, EC low-dose, and EC high-dose significantly reduced the serum levels of IL-6 (*P* < 0.01, respectively) and IL-1*β* (*P* < 0.01, *P* < 0.05, *P* < 0.01, respectively); compared to the model group, the interventions of positive control drug and EC high-dose significantly decreased the serum level of IL-1*β* in the model group (*P* < 0.01, respectively) (Figure 3(a)). Meanwhile, the mRNA expressions of proinflammatory factors *IL-6*, *IL-1β*, and *TNF-α* in the liver tissue were detected using qPCR. Compared to the control group, the mRNA expressions of *IL-6, IL-1β,* and *TNF-α* in the liver were significantly upregulated in the model group (*P* < 0.01, respectively, Figure 3(b)); while the mRNA levels of *IL-6* (*P* < 0.01, *P* < 0.05, *P* < 0.01, respectively) and *IL-1β* (*P* < 0.01, *P* < 0.05, *P* < 0.01, respectively) in simvastatin, EC low-dose, and EC high-dose were significantly downregulated compared to those in the model group. Compared to the model group, the intervention of simvastatin and high-dose EC significantly reduced mRNA expression of *TNF-α* in the liver (*P* < 0.01, respectively) (Figure 3(b)).

The effects of EC on oxidative stress in rats in the NAFLD model group was evaluated by further measuring SOD, MDA, and GSH-Px levels in the liver tissue homogenates of each group. Compared to the control group, SOD and GSH-Px activities in liver tissue homogenates of rats in the model group were significantly reduced (*P* < 0.01, respectively), and MDA levels were significantly increased (*P* < 0.01, respectively). The activity of SOD in the liver homogenate was increased in simvastatin, EC low-dose, and EC high-dose groups compared with the model group (*P* < 0.01, respectively). Compared to the model group, simvastatin and high-dose of EC treatment increased the activity of GSH-Px (*P* < 0.05, *P* < 0.01, respectively) and decreased the level of MDA (*P* < 0.01, respectively) in the liver homogenate (Table 2).

Altogether, EC high-dose had a significant therapeutic effect on NAFLD rats, while significantly decreasing the inflammatory response and enhancing oxidative stress in the liver tissue; therefore, the EC high-dose group was selected for subsequent experiments to study the effects of EC on 16S rRNA sequencing and serum metabolite levels in NAFLD rats.

### 3.3. Effect of EC on Gut Microbiota of NAFLD Rats

**Figure 4:**
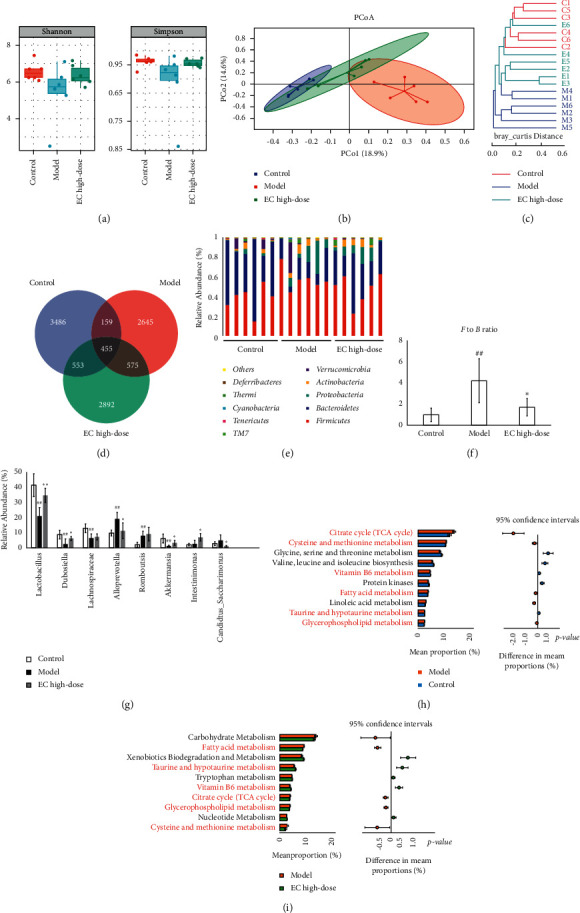
EC treatment affected the gut microbiota community in NAFLD model rats. (a) There were no significant differences in Shannon and Simpson indexes among each group. (b, c) PCoA and system clustering tree indicated that beta diversity of gut microbiota between EC high-dose and control groups was more similar than that between the model and control groups (“C” indicates control group; “M” indicates model group; “E” indicates EC high-dose group). (d) The different numbers of OTUs are shown using Venn diagram. (e, f) EC treatment reduced the F to B ratio in gut microbiota at the phylum level. (g) At the genus level, EC treatment affected the relative abundances of *Lactobacillus*, *Dubosiella*, *Desulfovibrio*, *Romboutsis, Intestinimonas* and *C._saccharimonas* in NAFLD rats. (h, i) The differential metabolic pathways (written in red) of EC on NAFLD were predicted using PICRUSt analysis based on the 16S rRNA sequencing data. Control, model, and EC high-dose (*n* = 6 per group) groups are shown. Data are reported as the mean ± SD. ^##^*P* < 0.01 compared with the control group; ^*∗*^*P* < 0.05 compared with the model group; ^*∗∗*^*P* < 0.01 compared with the model group.

To investigate the changes of EC on the gut microbiota of NAFLD rats, the fecal microbiota of different groups of rats were analyzed using 16S rRNA high-throughput sequencing. Shannon and Simpson indexes were measured to evaluate the alpha diversity of gut microbiota in each group. There were no significant differences in Shannon and Simpson indexes among each group (Figure 4(a)).

Next, the composition of microbial communities of different samples was analyzed using *ß*-diversity and evaluated using principal coordinate analysis (PCoA) and clustering analysis. The PCoA results showed that the sample points of the model group could be completely separated from those in the control group, whereas the sample points of the EC high-dose group were very close to those in control group (Figure 4(b)). Likewise, clustering analysis indicated that the distance from the control group to the EC high-dose group was closer than the distance from the normal group to the model group (Figure 4(c)).

To further explore the *α*-diversity and *ß*-diversity in the composition of gut microbiota, the number of common and unique OTUs among various groups was analyzed using Venn diagram to visualize the similarity and overlap in OTU number composition among the samples. The results are indicated in the figure, where it was found that the number of OTUs common to the normal group, the model group, and the EC high-dose group were 455 species, while the number of OTUs unique to these three groups were 3,486 species, 2,645 species, and 2,892 species, respectively (Figure 4(d)).

The composition of the gut microbiota in each sample at the phylum level is shown in Figure 4(e). *Firmicutes* and *Bacteroidetes* were dominant taxa at the phylum level. Compared with the control group, the *Firmicutes*/*Bacteroidetes* (F/B) ratio was increased in the model group (*P* < 0.01). Compared with the model group, the F/B ratio in the EC high-dose group was decreased (*P* < 0.05, Figure 4(f)). At the genus level, compared to the normal group, the model group had relatively higher abundance of *Desulfovibrio*, *Romboutsia*, and *Lactobacillus*, *Dubosiella*, *Lachnospiraceae*, and *Akkermansia* had relatively lower abundance; compared to model group, *Lactobacillus*, *Dubosiella*, *Lachnospiraceae*, *Akkermansia*, and *Intestinimonas* had relatively higher abundance in the EC high-dose group, and the abundance of *Desulfovibrio* and *C._saccharimonas* was relatively lower (Figure 4(g)).

### 3.4. Functional Prediction of 16S rRNA Sequencing Based on PICRUSt Analysis

PICRUSt analysis was further used to investigate the functional changes in the gut microbiota of NAFLD rats after EC high-dose treatment. Figures 4(e) and 4(f) indicated the top 10 metabolic pathways with the highest proportion and *P* < 0.05. Pathways that were changed in the normal and model groups also in the model and EC groups were considered differential metabolic pathways. Compared to the control group, the abundance of citrate cycle (TCA cycle), cysteine and methionine metabolism, and fatty acid metabolism increased in the model group. In contrast, the abundance of vitamin B6 metabolism reduced; compared with the model group, the abundance of fatty acid metabolism, TCA cycle, glycerophospholipid metabolism, cysteine, and methionine metabolism decreased in the EC high-dose group, but in contrast, the abundance of taurine and hypotaurine metabolism, and vitamin B6 metabolism improved (Figures 4(h) and 4(i)).

### 3.5. Changes in Serum Metabolite Levels in NAFLD Rats after EC Treatment

**Figure 5:**
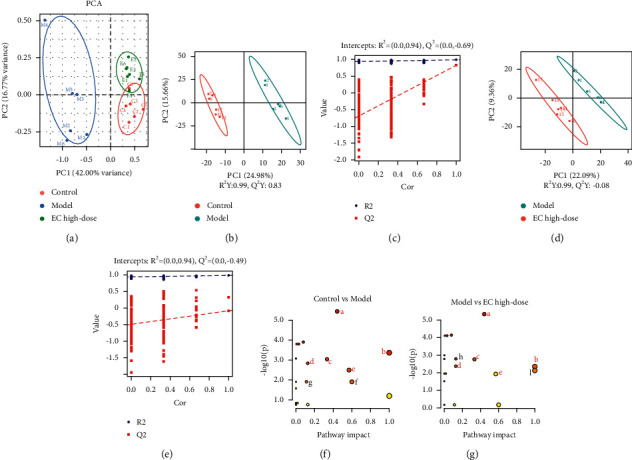
EC modulated the metabolites in serum in NAFLD rats. (a) PCA among groups. (b, c) OPLS-DA of untargeted metabolomics data between the control and model groups and the relative coefficient of loading plots. (d, e) OPLS-DA untargeted metabolomics data between the model and EC high-dose groups and the relative coefficient of loading plots. (f, g) Pathway analysis of differential metabolites between control and model groups (f) and between model and EC high-dose groups (g). The common pathways were written in red. a, arachidonic acid metabolism; b, taurine and hypotaurine metabolism; c, alpha-linolenic acid metabolism; d, cysteine and methionine metabolism; e, vitamin B6 metabolism; f, synthesis and degradation of ketone bodies; g, butanoate metabolism; h, sulfur metabolism; i, linoleic acid metabolism.

The PCA indicated that the control group could be well differentiated from the model group, and the model group was well differentiated from the EC high-dose group (Figure 5(a)). Furthermore, OPLS-DA model was used to identify the differential metabolites (Figures 5(b) and 5(c)). Compared with the control group, model group had explanatory rate (*R*^2^) = 0.94 and predictive power (*Q*^2^) = –0.60; compared to the model group, EC high-dose group had *R*^2^ = 0.94 and *Q*^2^ = –0.49 (Figures 5(d) and 5(e)). These results showed that the model was stable and had a good predictive ability.

The following two criteria were used to screen the differential metabolites: *P* < 0.05 and VIP >1.0, and 21 differential metabolite was identified (Table 3). Compared to the control group, the serum levels of acetoacetate, cholesterol, AA, 4-pyridoxic acid, stearic acid, taurocholic acid, prostaglandin G2, 16(R)-HETE, methionine, taurine, and pyridoxic acid were significantly improved in the model rats, while the levels of methionine, taurine, pyridoxal, L-cystine, hypotaurine, pyridoxamine, L-cysteinesulfinic acid, cholecalciferol, and *α*-linolenic acid were significantly reduced; compared to the model group, the serum levels of methionine, taurine, L-cystine, pyridoxamine, L-cysteinesulfinic acid, linoleic acid, calcitriol, cholecalciferol, and *α*-linolenic acid were significantly increased, while the levels of cholesterol, AA, APS, palmitic acid, 4-pyridoxic acid, taurocholic acid, prostaglandin G2, and 16(R)-HETE were significantly reduced (Table 3).

### 3.6. Analysis of Differential Metabolic Pathways in the Serum after EC Intervention in NAFLD Rats

The MetaboAnalyst analysis platform was used to conduct the enrichment analysis of the metabolic pathway for differential metabolites, and KEGG was selected as the database to screen the differential metabolic pathways based on the condition of pathway impact >0.1 and *P* < 0.05. The differential metabolic pathways between control and model groups included taurine and hypotaurine metabolism, AA metabolism, alpha-linolenic acid metabolism, cysteine and methionine metabolism, vitamin B6 metabolism, synthesis and degradation of ketone bodies, and butanoate metabolism (Figure 5(f)). The differential metabolic pathways between model and EC high-dose groups included taurine and hypotaurine metabolism, AA metabolism, alpha-linolenic acid metabolism, cysteine and methionine metabolism, vitamin B6 metabolism, sulfur metabolism, linoleic acid metabolism (Figure 5(g)). Among them, AA metabolism, taurine and hypotaurine metabolism, alpha-linolenic acid metabolism, cysteine and methionine metabolism, and vitamin B6 metabolism pathways were common between the normal and model groups, and also between the model and EC high-dose groups, where these pathways were selected to be the metabolic pathways of the EC intervention model group.

### 3.7. Correlation Analysis of Nontargeted Metabolomics and Gut Microbiota

**Figure 6:**
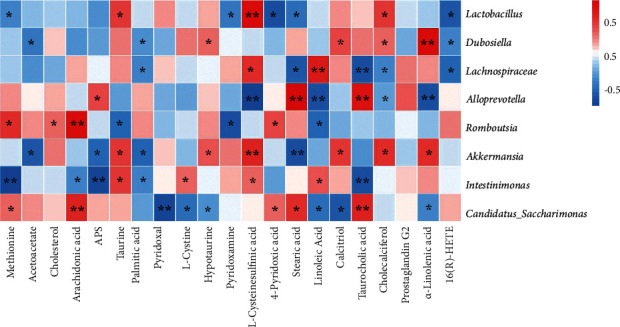
Spearman correlation analysis of gut microbiota with differential abundance and differential metabolites in the serum (heatmap). Red grids indicate positive correlations between gut microbiota and metabolites (correlation analysis value > 0.1), and blue grids indicate negative correlations between gut microbiota and metabolites (correlation analysis value < −0.1). ^*∗*^*P* < 0.05; ^*∗∗*^*P* < 0.01.

Spearman's correlation analysis was used to assess the relationship between serum differential metabolites and genus levels of gut microbiota in the normal, model, and EC high-dose groups. As indicated in Figure 6, *Lactobacillus*, *Dubosiella*, *Lachnospiraceae*, *Desulfovibrio*, *Romboutsia*, *Akkermansia*, *Intestinimonas*, and *C._saccharimonas* were correlated with most of the metabolites.

### 3.8. Correlation Analysis of Therapeutic Effect Indicators, Oxidative Stress Indicators, and Proinflammatory Cytokines with Gut Microbiota

**Figure 7:**
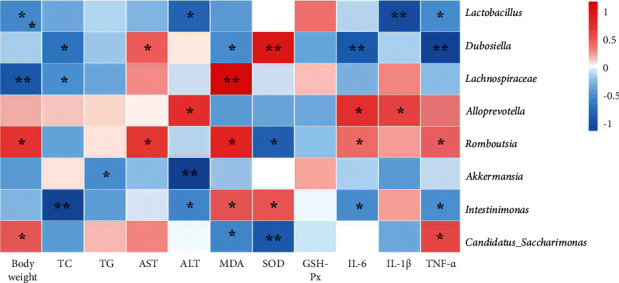
Spearman correlation analysis between general statement, biochemical markers, and proinflammatory cytokines and gut microbiota (heatmap). Red grids indicate positive correlations between general statement, biochemical markers, and proinflammatory cytokines and gut microbiota (correlation analysis value > 0.1), while blue grids indicate negative correlations between general statement, biochemical markers, and proinflammatory cytokines and gut microbiota (correlation analysis value < −0.1). ^*∗*^*P* < 0.05; ^*∗∗*^*P* < 0.01.

Spearman correlation analysis was used to assess the relationship between therapeutic effect indicators, oxidative stress levels, proinflammatory cytokines, and gut microbiota in the control, model, and EC high-dose groups. As indicated in Figure 7, *Dubosiella*, *Lactobacillus*, and *Intestinimonas* were negatively correlated with most of the physiological indicators, and *Romboutsia* and *Desulfovibrio* were positively correlated with most of the physiological indicators. In addition, *Dubosiella*, *Lactobacillus*, and *Intestinimonas* were negatively correlated with certain proinflammatory cytokines, and *Romboutsia* and *Desulfovibrio* were positively correlated with certain proinflammatory cytokines. *Dubosiella*, *Lachnospiraceae*, *Romboutsia*, *Intestinimonas*, and *C._saccharimonas* were correlated with certain oxidative stress factors.

## 4. Discussion

In this study, high-fat chow feeding was used to generate the NAFLD rat model, which is consistent with the previous study [31]. Compared to the control group, rats in the model group had significantly increased body weight and serum levels of TG, TC, ALT, and AST. Pathological examination also indicated significant steatosis and cellular damage in hepatocytes of rats in the model group, which is consistent with pathological NAFLD manifestations. Each EC dose group could decrease body weight, improve dyslipidemia, and alleviate liver pathological changes in NAFLD rats to different degrees, suggesting that EC has a therapeutic effect on NAFLD, and is most evident in the high-dose group. Simvastatin is a first-line regimen for treating NAFLD in clinical practice, which decreases body weight and increases serum ALT levels in patients with NAFLD [32]. It enhances NAFLD by increasing endothelial nitric oxide synthase (eNOS) expression, reducing inducible nitric oxide synthase (iNOS) expression, and inhibiting hepatic stellate cell (HSC) activation [33]. Therefore, simvastatin was chosen as the positive control group. The results indicated that simvastatin did not vary significantly from the EC high-dose group in improving body weight, blood lipids, and pathological changes.

Then, we evaluated the effect of EC on the level of oxidative stress and inflammation in rats with NAFLD, which has a complex pathogenesis, but the “two-hit” hypothesis is widely accepted. Fatty acids enter the liver and are deposited as TG in huge amounts in the liver parenchyma cells, and the gradual disruption of intracellular metabolism is the first hit of the disease. When cells are unable to store large amounts of free fatty acids as TG or exceed the load of cellular oxidative, the generation of large number of reactive oxygen species (ROS) from excess fatty acids inducing endoplasmic reticulum stress, oxidative stress, apoptosis, and inflammatory responses is the second hit to disease onset [34]. Our results discovered that EC could increase SOD and GSH-Px activities and reduce MDA levels in NAFLD rats, suggesting that EC can decrease oxidative stress in NAFLD model rats. As the end product of lipid peroxidation under oxidative stress, level of MDA could reflect the severity of oxidative damage in cells and organs [35]. SOD acts as an intracellular oxygen radical scavenger, catalyzing O_2_^–^ to O_2_ and H_2_O_2_ and protects the body from superoxide anions [36, 37]. GSH-Px catalyzes the conversion of glutathione (GSH) to oxidized glutathione (GSSG) and protects cells from oxidative stress [38].

ELISA and qPCR results indicated that EC could reduce the inflammatory response, as evidenced by the downregulation of gene expression of liver *IL-1β*, *IL-6*, and *TNF-α* with the decrease IL-1*β*, IL-6, and TNF-*α* levels in serum. Excessive lipid levels cause hepatocytes to produce proinflammatory factors such as IL-6, IL-1*β*, and TNF-*α*, causing NAFLD onset [39, 40]. TNF-*α* is closely related to the body's inflammatory response, lipid metabolism, and cell death [41]. IL-6 is also an essential proinflammatory factor that induces liver injury, causes hepatocyte apoptosis, manufactures insulin resistance, and is involved in the development and progression of NAFLD [42]. IL-1*β* is involved in the metabolic activity of different acute liver injury symptoms and promotes hepatic steatosis [43]. These inflammatory cytokines cause compensatory hyperinsulinemia, which impairs hepatic fat metabolism and causes hepatocellular steatosis; therefore, the suppression of the inflammatory response can alleviate NAFLD [44].

The effect of the EC high-dose group on the gut microbiota of NAFLD model rats was investigated using 16S rRNA sequencing technology. The Simpson index and Shannon index reduced in the model group and increased after EC administration, proposing that EC could increase the alpha diversity of gut microbiota in NAFLD rats. The results of PCoA plot and clustering tree indicated that the beta diversity of the gut microbiota of rats in the EC group was more similar to that of the control group than to that of the model group, and these results suggested that EC could restore the beta diversity of the gut microbiota of NAFLD rats to control group's level. Further analysis of microbiota abundance indicated that at the phylum level, the main gut microbiota species in each group were *Bacteroidetes* and *Firmicutes*, and the ratio of *Firmicutes* to *Bacteroidetes* was increased in the NAFLD model rats, while EC significantly decreased the F/B ratio of gut microbiota. The F/B ratio is closely related to the inflammatory response of the organism and dyslipidemia [45]. Compared to healthy controls, patients with dyslipidemia had a significant increase in the relative abundance of *Firmicutes*, a significant reduction in *Bacteroidetes*, and an increase in the ratio [46]. Clinically, decreasing the F/B ratio in patients enhances lipids [47].

Further analysis at the genus level indicated that the abundance of *Lactobacillus*, *Dubosiella*, *Lachnospiraceae*, and *Akkermansia* was significantly reduced, and the abundance of *Alloprevotella* and *Romboutsia* was significantly increased in the model rats compared to that in the control group. EC treatment increased the abundance of *Lactobacillus*, *Dubosiella*, *Akkermansia*, and *Intestinimonas* and reduced the abundance of *Alloprevotella* and *C._saccharimonas* in NAFLD model rats. *Lactobacillus* is a major probiotic with an essential role in regulating body metabolism immunity, and so on [48–50]. The abundance of *Lactobacillus* was decreased in models of diabetes, fatty liver, and obesity, and the metabolic and inflammatory responses of the body could be enhanced by transplanting *Lactobacillus* [51]. The *Dubosiella* was decreased in mice with acute alcoholic liver injury [52]. The *Dubosiella* are potential beneficial bacteria for colitis [53], and studies have indicated that the mechanism of action of Chinese medicinal plant extracts from *Lonicera hypoglauca* and *Scutellaria baicalensis* in alleviating inflammation and oxidative stress in mice with colitis may be related to increased *Dubosiella* abundance [54]. The *Lachnospiraceae* are butyrate-producing bacteria, and one study found that the abundance of *Lachnospiraceae* was decreased in patients with NAFLD [55]. Butyrate production by bacteria is a key function in maintaining gut microbiota homeostasis, and butyrate significantly alleviates steatohepatitis by regulating gut microbiota and intestinal barrier function, thereby decreasing inflammation and oxidative damage in the liver [56]. *Desulfovibrio* promotes inflammatory responses and metabolic disorders in the organism. In a type 2 diabetes model, the abundance of *Desulfovibrio* was significantly elevated [57]. Suppression of *Desulfovibrio* abundance by microbiota transplantation may improve diabetes [58]. *Romboutsia* are bacteria associated with obesity, and some studies have indicated a positive correlation between *Romboutsia* and body fat [59]. In a high-fat diet-induced hyperlipidemia rat model, spirulina platensis 55% ethanol extract enhanced lipid metabolism by reducing the abundance of *Romboutsia* [60]. The increased abundance of *Akkermansia* decreases the elevated lipopolysaccharide levels induced by the high-fat diet and is essential for maintaining homeostasis of glucose metabolism [61]. One study found a significant increase in the percentage of *Akkermansia* in the gut microorganisms of obese mice treated with metformin and its role in inhibiting lipid metabolic processes [62]. *Intestinimonas* are also butyrate-producing enterobacteria with antiobesity and anti-inflammatory effects [63]. *C._saccharimonas* are conditionally pathogenic bacteria that are significantly elevated in the intestine of gout patients [64]. In addition, green tea leaf powder reduces high-fat diet-induced disorders in lipid metabolism, while reducing the abundance of *C._saccharimonas* in their intestines [65]. PICRUSt analysis was used to predict the metabolic pathways associated with altered gut microbiota, including fatty acid metabolism, taurine and hypotaurine metabolism, vitamin B6 metabolism, TCA cycle, glycerophospholipid metabolism, and cysteine and methionine metabolism.

PCA and OPLS-DA of serum nontargeted metabolomics indicated that the metabolites of NAFLD model rats significantly varied from that of the control rats, and the serum metabolite levels of rats after EC intervention significantly varied from those of NAFLD model rats. The metabolic pathway analysis of the differential metabolites using MetaboAnalyst indicated that EC could affect the metabolic pathways of AA metabolism, taurine and hypotaurine metabolism, alpha-linolenic acid metabolism, cysteine and methionine metabolism, and vitamin B6 metabolism. The taurine and hypotaurine metabolism, cysteine and methionine metabolism, and vitamin B6 metabolism pathways were common pathways, proposing that EC may affect NAFLD treatment by regulating gut microbiota, which in turn affects the pathways of taurine and hypotaurine metabolism, cysteine and methionine metabolism, and vitamin B6 metabolism.

### 4.1. Taurine and Hypotaurine Metabolism

Taurine and hypotaurine metabolism are closely related to many diseases. In our study, it was found that taurine and hypotaurine levels were decreased in NAFLD model rats and were significantly increased after EC treatment. Taurocholic acid levels were increased and decreased after EC treatment. Taurine has different physiological functions in cells, such as improving glucolipid metabolism, immunomodulation, anti-inflammation, and antioxidation [66]. It has been shown that taurine supplementation prevents obesity and insulin resistance [67]. Taurine can decrease inflammation in adipose tissue by decreasing macrophage infiltration [68]. In addition, hepatic taurine levels affect hepatic steroid metabolism and other lipid metabolism [69–71]. Taurine is a recognized antioxidant and increases the activity of the antioxidant enzymes superoxide dismutase and glutathione peroxidase [72]. Ebrahim and Sakthisekaran showed that taurine supplementation in rats was effective in avoiding biomembrane damage induced by lipid peroxidation by increasing the antioxidant capacity of the body [73]. High concentrations of taurine may help protect the gastric mucosa from oxidative stress [74]. Taurine has a hepatoprotective effect and was found to protect against acetaminophen-induced liver injury in rats, and taurine also enhanced many liver function indicators in a carbon tetrachloride-induced liver fibrosis model by improving serum transaminase and bilirubin levels and antioxidant effects [75, 76]. Hypotaurine is a precursor of taurine synthesis that produces taurine by enzymatic action [77]. It is an antioxidant and singlet oxygen scavenger that protects cells from oxidative stress damage [78]. It has been proposed that the antioxidant effect of hypotaurine may be related to the prevention of SOD enzyme inactivation [79]. Hypotaurine decreased liver injury and fat accumulation in the NAFLD mice model, and hypotaurin supplementation was shown to increase insulin sensitivity in obese mice in an obesity model [79]. Taurocholic acid is a conjugated primary bile acid formed by bile acid binding to taurine in hepatocytes. In cholestatic liver disease, taurocholic acid-induced inflammatory response plays an essential role in disease progression [80]. Taurocholic acid levels are significantly elevated in the metabolic profile of patients with NAFLD [81]. Taurocholic acid in the liver activates sphingosine 1-phosphate receptor 2 (S1PR2)-mediated signaling pathways involved in the process of liver inflammation and fibrosis [82]. Spearman's analysis indicated that taurine was associated with *Lactobacillus*, *Akkermansia*, and *Intestinimonas*, and negatively correlated with *Romboutsia*; hypotaurine was positively correlated with *Dubosiella* and *Akkermansia* and negatively correlated with *C._saccharimonas*; and taurocholic acid was positively correlated with *Desulfovibrio* and *C._saccharimonas and* negatively correlated with *Lachnospiraceae* and *Intestinimonas.* Therefore, it was speculated that the effect of EC on taurine and hypotaurine metabolism may be related to the regulation of the abundance of *Lactobacillus*, *Akkermansia, Intestinimonas*, *Romboutsia, Dubosiella, Lachnospiraceae, Desulfovibrio,* and *C._saccharimonas*.

### 4.2. Cysteine and Methionine Metabolism

Cysteine and methionine metabolism is an amino acid metabolism closely related to oxidative stress and can regulate oxidative stress through the methionine/glutathione S-transferase pathway [83]. Our study found that methionine, L-cystine, and L-cysteinesulfinic acid were reduced in model group rats and increased after drug administration. Methionine and choline deficiency diet is commonly used to cause NAFLD in rodents. Methionine deficiency decreases in mice low density lipoprotein (LDL) synthesis and dysfunction of mitochondrial *ß*-oxidation, causing rapid and massive accumulation of triacylglycerol in hepatocytes, cause hepatocyte steatosis, which constitutes the “first hit” and increases the susceptibility of the liver to other injuries [84]; hepatocyte steatosis leads to decreased mitochondrial uptake of free fatty acids, contributing to increased free fatty acid *ß*-oxidation, causing oxidative stress and lipid peroxidation, constituting a “second hit” to the liver [85]. Methionine supplementation to a high-cholesterol diet can significantly decrease hepatic steatosis, oxidative stress, and fibrosis induced by high choline [86]. Methionine is one of the important amino acids in the body, providing the methyl group required for the synthesis of choline in the body, which promotes phospholipid synthesis, synthesizes lipoproteins, and facilitates the transport of fat to tissues other the liver to alleviate fatty liver [87, 88]. L-cystine is a precursor of the antioxidant GSH and an essential nutrient for maintaining normal liver function. L-cystine can be converted from methionine to N-acetylcysteine (NAC) and taurine, among others, to supply the needs of cells [89, 90]. Supplementation with L-cystine reduces the elevated serum triglyceride and TC concentrations induced by liver cancer [91]. L-cystine can be converted to L-cysteine by the action of cysteine reductase [92]. Cysteine is converted to decreased glutathione (GSH) catalyzed by glutamate cysteine ligase (GCLC) and glutathione synthetase (GSS), which is involved in the decrease process of cells and phospholipid metabolism in the liver, protecting cells from lipid peroxidation damage [93]. It has been indicated that L-cysteine can upregulate GSH levels and improve oxidative stress in a high-glucose cultured hepatocyte model and in diabetic fatty liver rats [94]. Long-term supplementation with L-cysteine restores cardiovascular lesions caused by a high-fat diet in rats [95]. Dietary supplementation with L-cysteine dose-dependently promoted the antioxidant enzyme activity and decreased lipid levels in the serum and rat liver [96]. L-cysteinesulfinic acid is an intermediate in the *in vivo* oxidation of L-cystine and L-cysteine and is a precursor of taurine. Cysteine sulfinate is decarboxylated to hypotaurine by cysteine sulfinate decarboxylase, and then oxidized to taurine. Spearman's analysis indicated that methionine was positively correlated with *Romboutsia* and *C._saccharimonas* and negatively correlated with *Lactobacillus* and *Intestinimonas*; L-cystine was positively correlated with *Intestinimonas* and negatively correlated with *C._saccharimonas*; and *L-cysteinesulfinic acid* was positively correlated with *Lactobacillus*, *Lachnospiraceae*, *Akkermansia*, and *Intestinimonas*, and negatively correlated with *Desulfovibrio.* Therefore, it was speculated that the effect of EC on cysteine and methionine metabolism may be related to the regulation of the abundance of *Lactobacillus*, *Lachnospiraceae*, *Akkermansi*, *Intestinimonas*, *Romboutsia*, *Desulfovibrio*, and *C._saccharimonas*.

### 4.3. Vitamin B6 Metabolism

Vitamin B6 metabolism has antioxidant, hepatoprotective, blood cholesterol lowering, and anti-angiosclerosis effects [97, 98]. In our study, it was found that pyridoxal and pyridoxamine were significantly reduced in the model rats and increased after the administration of EC, while 4-pyridoxic acid was increased in the model rats and reduced after drug administration. Pyridoxal is an active form of vitamin B [99], and pyridoxal is normally converted to pyridoxal-5-phosphate (PLP) by pyridoxal kinase, which acts as a cofactor for several enzymes involved in amino acid metabolism [100]. PLP can attenuate calcium signaling *in vitro* and inhibit calcium entry into adipocytes and subsequent expression of adipocyte fatty acid synthase (FASN), thereby inhibiting lipid synthesis [101–103]. PLP treatment increased fat oxidation and insulin sensitivity in 3T3L1 adipocytes and significantly decreased oxidative and inflammatory stress [104]. Pyridoxamine is a possible inhibitor of advanced glycation end products, and is a vitamin B6 derivative and anti-glycation agent [105]. A study has indicated that pyridoxamine significantly inhibits the expression of proinflammatory genes in visceral adipose tissue of HFD mice [106]. Pyridoxamine inhibits HFD-induced weight gain and macrophage M1 polarization in obese rats and increases GLO1 expression in perivascular and visceral adipose tissue through the RAGE pathway, where it was speculated that pyridoxamine is a candidate for treating obesity or obesity-related inflammatory complications [107]. Pyridoxamine modulates oxidative stress, advanced glycosylation, inflammatory factor levels, and metabolic disorders. Also, it improves microcirculation in patients with NAFLD [108]. The 4-pyridoxic acid is the final vitamin B6 breakdown product. Vitamin B6 is metabolized to PLP in the body, which is further dephosphorylated and oxidized, and eventually generates 4-pyridoxic acid, excreted in the urine [109]. Some studies have indicated that serum 4-pyridoxic acid is elevated in renal insufficiency [110]. However, the relationship between 4-pyridoxic acid and fatty liver is unclear and requires studying in subsequent experiments. Spearman analysis indicated that pyridoxal was negatively correlated with *C._saccharimona*; pyridoxamine was negatively correlated with *Lactobacillus* and *Romboutsia*; and 4-pyridoxic acid was positively correlated with *Romboutsia* and *C._saccharimona* and negatively correlated with *Lactobacillus*. Therefore, it was speculated that the effect of EC on the TCA cycle may be related to the regulation of the abundance of *C._saccharimonas*, *Lactobacillus,* and *Romboutsia.*

## 5. Conclusions

In conclusion, our study showed multiple ameliorative effects of EC on NAFLD, including enhancement of liver function and blood lipids, as well as alleviation of pathological changes, oxidative stress, and inflammatory responses. The mechanism of EC for NAFLD may be related to the enhancement of gut microbiota alteration, regulation of metabolic pathways of taurine and hypotaurine metabolism, and cysteine and methionine metabolism as well as vitamin B6 metabolism.

## Figures and Tables

**Table 1:** Changes in blood lipid levels and liver enzymes after EC treatment.

Group	TC (mmol/L)	TG (mmol/L)	ALT (U/L)	AST (U/L)
Control	9.48 ± 2.01	2.74 ± 0.70	28.32 ± 9.28	40.36 ± 28.87
Model	22.92 ± 8.91^##^	5.30 ± 1.34^##^	58.58 ± 26.29^##^	176.41 ± 46.42^##^
Simvastatin	10.27 ± 3.46^*∗∗*^	2.14 ± 0.59^*∗∗*^	35.16 ± 17.42^*∗*^	87.30 ± 28.64^*∗∗*^
EC low-dose	19.94 ± 8.85	5.02 ± 2.12	54.48 ± 19.18	143.90 ± 30.48
EC high-dose	12.78 ± 6.02^*∗*^	2.75 ± 0.91^*∗∗*^	38.00 ± 12.48^*∗*^	67.29 ± 28.06^*∗∗*^

Control, model, simvastatin, EC low-dose, and EC high-dose (*n* = 10 per group) groups are shown. Data are presented as the mean ± SD. ^##^*P* < 0.01 as compared to the control group; ^*∗*^*P* < 0.05 as compared to the model group; ^*∗∗*^*P* < 0.01 as compared to the model group. TC: total cholesterol; TG: triglyceride; ALT: alanine aminotransferase; AST: aspartate aminotransferase.

**Table 2:** The activities of SOD and GSH-Px and the level of MDA in the rat liver homogenate after EC treatment.

Group	SOD (U/mgprot)	MDA (nmol/mgprot)	GSH-px (U/mgprot)
Control	173.07 ± 18.56	3.68 ± 0.85	96.56 ± 9.73
Model	106.52 ± 30.12^##^	15.95 ± 2.43^##^	61.75 ± 13.33^##^
Simvastatin	161.24 ± 23.56^*∗∗*^	8.39 ± 3.01^*∗∗*^	79.63 ± 18.79^*∗*^
EC low-dose	144.82 ± 21.48^*∗∗*^	13.72 ± 3.00	68.75 ± 14.48
EC high-dose	149.78 ± 33.08^*∗∗*^	9.84 ± 4.20^*∗∗*^	79.50 ± 5.76^*∗∗*^

Control, model, simvastatin, EC low-dose, and EC high-dose (*n* = 10 per group) groups are shown. Data are presented as the mean ± SD. ^##^*P* < 0.01 as compared to the control group; ^*∗*^*P* < 0.05 as compared to the model group; ^*∗∗*^*P* < 0.01 as compared to the model group. SOD: superoxide dismutase; MDA: methane dicarboxylic aldehyde; GSH-Px: glutathione peroxidase.

**Table 3:** The differential metabolites in the serum of NAFLD rats.

No.	Formula	RT (min)	m/z	Metabolites	VIP		FC		Trend		Pathway
					M vs. C	E vs. M	M vs. C	E vs. M	M vs. C	E vs. M	
1	C_5_ H_11_ N O_2_ S	2.00	150.06	Methionine	1.49	1.46	0.63	1.30	↓#	↑^*∗*^	d
2	C_4_ H_6_ O_3_	1.48	101.02	Acetoacetate	1.30	1.45	1.65	1.26	↑#	↑	F, g
3	C_27_ H_46_ O	15.18	387.36	Cholesterol	1.22	2.10	1.75	0.54	↑##	↓^*∗∗*^	
4	C_26_ H_54_ N O_7_ P	10.52	568.36	Arachidonic acid	1.69	1.28	1.81	0.65	↑##	↓^*∗∗*^	a
5	C_10_ H_14_ N_5_ O_10_ P S	5.32	426.01	APS	1.98	2.04	1.27	0.67	↑	↓^*∗∗*^	h
6	C_2_ H_7_ N O_3_ S	1.44	124.01	Taurine	1.59	1.85	0.46	1.89	↓##	↑^*∗*^	b
7	C_16_ H_32_ O_2_	10.43	255.23	Palmitic acid	2.35	1.27	1.31	0.67	↑	↓^*∗*^	
8	C_8_ H_9_ N O_3_	5.27	168.06	Pyridoxal	1.17	1.29	0.79	1.26	↓#	↑^*∗*^	e
9	C_6_ H_12_ N_2_ O_4_ S_2_	1.20	239.02	L-Cystine	1.44	1.00	0.68	1.42	↓##	↑^*∗*^	d
10	C_2_ H_7_ N O_2_ S	1.35	110.03	Hypotaurine	1.22	1.84	0.76	1.31	↓#	↑^*∗*^	b
11	C_8_ H_12_ N_2_ O_2_	1.24	169.09	Pyridoxamine	2.31	2.44	0.71	1.33	↓#	↑^*∗*^	e
12	C_25_ H_50_ N O_7_ P	14.64	566.35	L-Cysteinesulfinic acid	1.08	2.08	0.67	1.41	↓##	↑^*∗∗*^	b, d
13	C_8_ H_9_ N O_4_	3.73	184.06	4-Pyridoxic acid	1.60	1.98	1.60	0.60	↑#	↓^*∗∗*^	e
14	C_18_ H_36_ O_2_	11.32	283.26	Stearic acid	2.00	1.14	1.83	0.79	↑##	↓	
15	C_18_ H_32_ O_2_	7.72	279.23	Linoleic acid	1.23	2.82	1.31	1.48	↑	↑^*∗∗*^	i
16	C_27_ H_44_ O_3_	14.08	415.32	Calcitriol	1.60	1.52	1.38	1.56	↑	↑^*∗*^	
17	C_26_ H_45_ N O_7_ S	8.30	516.30	Taurocholic acid	1.76	1.61	1.44	0.66	↑##	↓^*∗∗*^	b
18	C_27_ H_44_ O	11.65	385.35	Cholecalciferol	1.61	1.85	0.60	1.57	↓##	↑^*∗*^	
19	C_20_ H_32_ O_6_	13.13	367.21	Prostaglandin G2	1.48	1.28	1.91	0.50	↑##	↓^*∗∗*^	a
20	C_18_ H_30_ O_2_	14.73	279.23	*α*-Linolenic acid	2.34	1.63	0.59	1.61	↓##	↑^*∗∗*^	c
21	C_20_ H_32_ O_3_	13.95	343.22	16(R)-HETE	1.79	2.68	1.87	0.51	↑##	↓^*∗∗*^	a

Control, model, and EC high-dose (*n* = 6 per group) groups are shown. ^#^*P* < 0.05 compared with the control group; ^##^*P* < 0.01 compared with the control group; ^*∗*^*P* < 0.05 compared with the model group; ^*∗∗*^*P* < 0.01 compared with the model group; ↑: increase; ↓: decrease; vs.: versus; C: control group; M: model group; E: EC high-dose group; RT: retention time; VIP: variable importance of projection; FC: fold change; a: arachidonic acid metabolism; b: taurine and hypotaurine metabolism; c: alpha-linolenic acid metabolism; d: cysteine and methionine metabolism; e: vitamin B6 metabolism; f: synthesis and degradation of ketone bodies; g: butanoate metabolism h: sulfur metabolism; i: linoleic acid metabolism.

## Data Availability

The raw data supporting the conclusions of this article will be made available by the corresponding authors, without undue reservation.
